# Choroïdopathie du lupus érythémateux systémique, à propos d'un cas

**DOI:** 10.11604/pamj.2013.14.119.1090

**Published:** 2013-03-28

**Authors:** Benatiya Andaloussi Idriss, Chraibi Fouad, Bhallil Salima, Abdellaoui Meryem, Tahri Hicham

**Affiliations:** 1 CHU Hassan II, Fès, Maroc

**Keywords:** Choroidopathie, lupus érythémateux systémique, choroidopathy, systemic lupus erythematosus

## Abstract

Le lupus érythémateux systémique (LES) est une maladie auto-immune multi systémique d’étiologie inconnue. Nous rapportons une manifestation très rare du LES représentée par une choroidopathie bilatérale sans hypertension artérielle associée. Il s'agit d'une patiente âgée de 45 ans, suivie pour un LES depuis 5 ans, qui présente une baisse de l'acuité visuelle bilatérale et progressive depuis 3 mois. L'examen du fond d’œil, complété l'angiographie à la fluorescéine et la tomographie en cohérence optique, retrouve un décollement séreux rétinien (DSR) multifocal et bilatéral. L’évolution après corticothérapie systémique est marquée par une amélioration de l'acuité visuelle et une régression du DSR. L'atteinte oculaire au cours du LED est dominée par la kérato-conjonctivite sèche (1/4 à 1/3 des cas). La choroïdopathie est plus rare: seulement une trentaine de cas sont rapportés dans littérature. Elle se manifeste par un décollement séreux rétinien et est habituellement observée chez les patients présentant une néphropathie ou une hypertension artérielle, ce qui n'est pas le cas de notre observation. L'apparition d'une atteinte oculaire au cours du LES peut annoncer une poussée d'atteinte systémique et doit susciter une enquête appropriée.

## Introduction

Le lupus érythémateux systémique (LES) est une maladie auto-immune multi systémique, aux manifestations cliniques très variées et caractérisée par la présence d′auto-anticorps anti-nucléaires. L'atteinte ophtalmologique est peu fréquente et reste dominée par la kérato-conjonctivite sèche. Nous rapportons une manifestation très rare du LES représentée par une choroidopathie bilatérale chez une patiente âgée de 45 ans sans hypertension artérielle associée.

## Patient et observation

**Figure 1:**
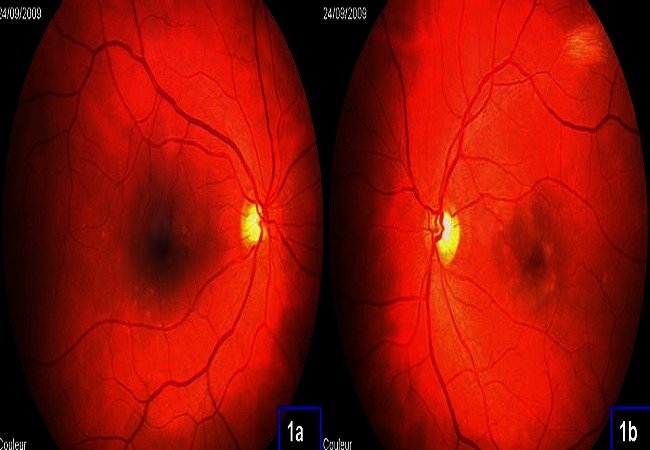
Aspect du fond d’œil de l’œil droit (1a) et de l’œil gauche (1b): remaniement maculaire bilatéral

**Figure 2:**
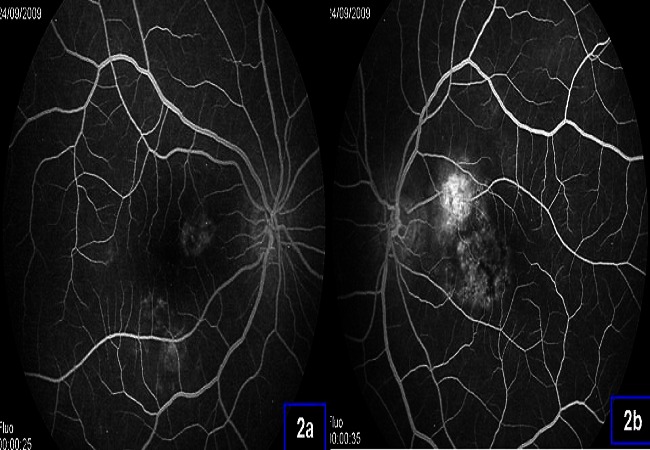
Aspect agiographique de l’œil droit (2a) et de l’œil gauche (2b): multiples zones hyper fluorescentes hétérogènes associées à de nombreuses hyperfluorescences en têtes d’épingle

Il s'agit d'une patiente âgée de 45 ans, suivie pour un LES depuis 5 ans, qui présente une baisse de l'acuité visuelle bilatérale et progressive depuis 3 mois. L'examen ophtalmologique retrouve une acuité visuelle avec correction réduite à 2/10 et P4 en OD et 6/10 et P3 en OG sans kérato-conjonctivite sèche ni signes d'uvéite antérieure ou postérieure. L'examen du fond d’œil montre aux deux yeux un remaniement maculaire bilatéral (Figure 1). L'angiographie à la fluorescéine retrouve en ODG de multiples zones hyper fluorescentes hétérogènes associées à de nombreuses hyerfluorescences en têtes d’épingle (Figure 2).

**Figure 3:**
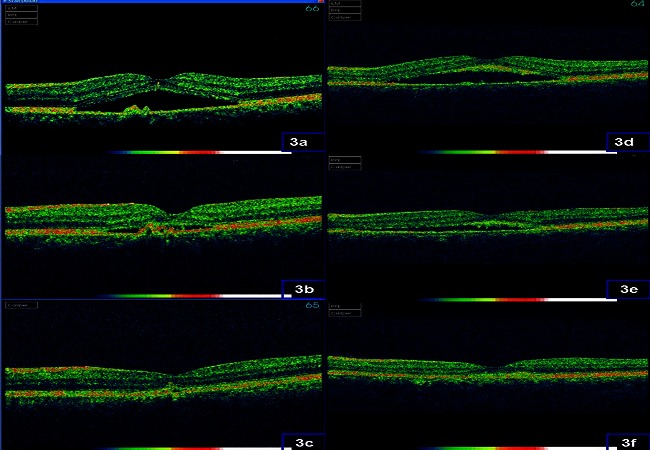
Aspects à la tomographie en cohérence optique: décollement séreux rétinien: de l’œil droit: (3a): avant traitement, (3b) à un mois du traitement, (3c) à 3 mois du traitement; de l’œil gauche: (3d): avant traitement, (3e) à un mois du traitement, (3f) à 3 mois du traitement.

L'OCT maculaire retrouve un décollement séreux rétinien multifocal et bilatéral (Figure 3). L'examen général montre un érythème malaire avec une tension artérielle normale. Le bilan biologique révèle des taux élevés d'anticorps anti-ADN natif et d'anticorps antinucléaires.

La patiente est mise alors sous corticothérapie systémique: bolus IV de solumédrol 1g/24h, puis un relai par voie orale pendant 1 mois à 1mg/kg/jour et dégression progressive sur 2 mois. L’évolution est marquée par une amélioration de l'acuité visuelle à 8/10 en ODG et une disparition du décollement séreux rétinien (Figure 3).

## Discussion

L'atteinte oculaire au cours du LED est dominée par la kérato-conjonctivite sèche, avec ou sans la xérostomie (1/4 à 1/3 des cas). D'autres atteintes peuvent exister [1, 2]: lésions palpébrales: à type d’éruption en aile de papillon ou de blépharite chronique; orbitopathie inflammatoire; uvéite antérieure, sclérite antérieure ou postérieure. Les autres atteinte neuro-ophtalmlogique: neuropathie optique: (rétrobulbaire, ischémique antérieure), paralysies oculomotrices, anomalies pupillaires, ophtalmoplégie internucléaire. Lésions rétiniennes et choroïdiennes incluent classiquement: une rétinopathie lupique se manifestant par des exsudats cotonneux avec ou sans hémorragies; plus rarement une occlusion de l'artère ou de la veine centrale de la rétine; exceptionnellement: une choroïdopathie comme c'est le cas de notre patiente. La choroïdopathie est plus rare: seulement 36 cas sont rapportés dans littérature entre 1968 et 2011 [3, 4, 5]. Elle est habituellement observée chez les patients gravement malades ou hypertendus [3, 5], ce qui n'est pas le cas de notre observation.

Sur le plan physiopathologique la choroïdopathie lupique est habituellement d'origine ischémique, observée chez les patients présentant néphropathie avec une hypertension artérielle et une vascularite systémique [3, 4, 5]. Plus rarement, elle est d'origine immunologique et serait due au dépôt de complexes immuns dans la choriocapillaire et à la présence d′auto-anticorps dirigés contre l′épithélium pigmentaire rétinien [3, 4]. Quel que soit le mécanisme, ces perturbations aboutissent à un décollement séreux de l′épithélium pigmentaire ou de la rétine.

En cas de poussée de la maladie lupique, en particulier avec néphropathie aigue sévère ou une vascularite du SNC, une atteinte choroïdienne doit être systématiquement recherchée et vice versa [4]. La choroïdopathie du lupus systémique peut précéder de plusieurs mois la poussée de la maladie et peut être un signe révélateur de la présence infraclinique d'une néphropathie réversible ou d'une neuropathie, permettant ainsi une prise en charge précoce avant que ces atteintes ne mettent en jeu le pronostic vital du patient [3, 4].

L'examen clinique complété par l'angiographie à la fluorescéine est largement suffisant pour faire le diagnostic de la choroïdopathie [4]. L′ICG montre un remplissage irrégulier avec des zones hypofuorescentes mal définies au temps précoces et une hyperfuorescence choroïdienne aux temps tardifs l'ICG peut détecter des lésions qui ne sont pas visibles à l′examen clinique ou à l'angiographie à la fluorescéine [6, 7, 8].

L'OCT permet de mieux analyser le siège de l'accumulation du liquide séreux en intra-rétinien, sous rétinien ou sous l'EP. Il reste un outil d′appoint utile pour le diagnostic et le suivi des patients atteints de la choroïdopathie, surtout en cas de néphropathie associée, où l'angiographie à la fluorescéine pourrait être nuisible [7, 9, 10]. Il est important d’éliminer d'autres diagnostics tels que la choriorétinopathie séreuse centrale surtout chez les patient sous corticothérapie, le syndrome de Vogt Koyanagi Harada, la coagulation intravasculaire disséminée, le purpura thrombotique thrombocytopénique et l'insuffisance rénale [7, 8].

Son traitement repose essentiellement sur la corticothérapie par voie systémique et locale avec recours fréquent au traitement immunosuppresseur en cas de cortico résistance [4].

## Conclusion

La présence de l'atteinte oculaire en particulier la choroïdopathie témoigne de la sévérité de la maladie lupique, et doit susciter une enquête appropriée avec indication précoce d'un traitement immunosuppresseur pour prévenir certaines atteintes pouvant mettre en jeu le pronostic vital des patients.

## References

[1] Sumit Dhingra, Panagiota Stavrou (2004). Indocyanine green angiography in systemic lupus erythematosus-associated uveitis. Ocul Immunol Inflamm..

[2] Russel W Read (2004). Clinical mini-review: systemic lupus erythematosus and the eye. Ocul Immunol Inflamm..

[3] Nirav V Kamdar, Amsalu Erko, Jason S Ehrlich, Jonathan W Kim, Neeraja Kambham, Glenn M Chertow (2009). Choroidopathy and kidney disease: a case report and review of the literature. Cases J..

[4] Nguyen QD, Uy HS, Akpek EK, Harper SL, Zacks DN, Foster CS (2000). Choroidopathy of systemic lupus erythematosus. Lupus..

[5] Daniele Hannouche, Jean-Francois Korobelnik, Isabelle Cochereau, Gilles Hayem, Johann Beaudreuil, Olivier Meyer, Thanh Hoang-Xuan (1995). Systemic lupus erythematosus with choroidopathy and serous retinal detachment. Int Ophthalmol..

[6] Magd Gharbiya, Francesco Bozzoni-Pantaleoni, Federico Augello, Corrado Balacco-Gabrieli (2002). Indocyanine green angiographic findings in systemic lupus erythematosus choroidopathy. Am J Ophthalmol..

[7] Sabine Kouprianoff, Christophe Chiquet, Laurence Bouillet, Jean-Paul Romanet (2010). OCT follow-up of systemic lupus erythematosus choroidopathy. Ocul Immunol Inflamm..

[8] Gharbiya M, Pecci G, Baglio V (2006). Indocyanine green angiographic fndings for patients with systemic lupus erythematosus nephropathy. Retina..

[9] Edouard S, Douat J, Sailler L, Arlet P, Astudillo L (2011). Bilateral choroidopathy in systemic lupus erythematosus. Lupus.

[10] Ozturk BT, Bozkurt B, Koyuncu Z, Kerimoglu H (2011). Follow-up of lupus choroidopathy with optical coherence tomography. Lupus.

